# Towards a Proper Assignment of Systemic Risk: The Combined Roles of Network Topology and Shock Characteristics

**DOI:** 10.1371/journal.pone.0077526

**Published:** 2013-10-17

**Authors:** Lasse Loepfe, Antonio Cabrales, Angel Sánchez

**Affiliations:** 1 Grupo Interdisciplinar de Sistemas Complejos, Departament of Mathematics, Universidad Carlos III de Madrid, Leganés, Madrid, Spain; 2 Department of Economics, University College London, London, United Kingdom; Semmelweis University, Hungary

## Abstract

The 2007-2008 financial crisis solidified the consensus among policymakers that a macro-prudential approach to regulation and supervision should be adopted. The currently preferred policy option is the regulation of capital requirements, with the main focus on combating procyclicality and on identifying the banks that have a high systemic importance, those that are “too big to fail”. Here we argue that the concept of systemic risk should include the analysis of the system as a whole and we explore systematically the most important properties for policy purposes of networks topology on resistance to shocks. In a thorough study going from analytical models to empirical data, we show two sharp transitions from safe to risky regimes: 1) diversification becomes harmful with just a small fraction (~2%) of the shocks sampled from a fat tailed shock distributions and 2) when large shocks are present a critical link density exists where an effective giant cluster forms and most firms become vulnerable. This threshold depends on the network topology, especially on modularity. Firm size heterogeneity has important but diverse effects that are heavily dependent on shock characteristics. Similarly, degree heterogeneity increases vulnerability only when shocks are directed at the most connected firms. Furthermore, by studying the structure of the core of the transnational corporation network from real data, we show that its stability could be clearly increased by removing some of the links with highest centrality betweeness. Our results provide a novel insight and arguments for policy makers to focus surveillance on the connections between firms, in addition to capital requirements directed at the nodes.

## Introduction

The increasing complexity and globalization of financial markets, together with excessive leverage, have been singled out jointly by the Financial Service Authority and the Financial Stability Board as major contributors to the financial crisis of 2007-2009 [1,2]. In response to this crisis the consensus among policymakers increased that a macro-prudential approach to regulation and supervision should be adopted. Macro-prudential regulation seeks to stabilize the financial system by taking into account risks arising from the interactions between financial institutions. Correspondingly, there is a clear demand from policy makers to the scientific community for new mathematical and computational tools that emphasize the analysis of crises rather than of calm periods [3], a context in which understanding the performance and vulnerabilities of economic networks stands out as a major challenge [4].

The currently preferred policy option is the regulation of capital requirements. The Basel III accords establish higher overall equity requirements and combat pro-cyclicality by forcing banks to build up an extra capital conservation buffer of 2.5% during good times. In the cross-sectional dimension, much effort is currently concentrated in the “too big to fail” (or better, “too central to fail” [5]) debate. This debate is about assessing the amount of systemic risk that can be attributed to an institution from its size and network position, and the amount of higher loss absorbency (HLA) that should be required from the systemically important financial institutions (SIFIs). Using an analogy from epidemics: tame the super-spreaders [6].

A related research strand focus on the effects of the network topology as a whole. The baseline discussion here is on the effects of network connectivity on its shock resistance. On one hand, higher interconnectedness can reduce the probability of default, as it allows adverse shocks to dissipate quicker [7,8]. Higher interconnectedness, on the other hand, results in larger effects once the shock size has crossed a critical threshold. In short, high interconnectedness provides “robust yet fragile” [9] properties to the network. Many other aspects of network properties have been studied, starting with the seminal work of Freixas et al. [10], who arranged firms in a star like manner (as opposed to complete networks), showing the effects of indirect exposure. Subsequent models focused on heterogeneity either in network structure (mainly degree distribution, assortativity and clustering, e.g. [11-14]) or in bank sizes [15]. In some cases the link between two factors was studied, such as heterogeneity in individual vulnerability versus shock size [16], or diversification versus level of capitalization [17,18]. 

On the other hand, most theoretical models of financial networks rely on extreme examples, such as complete networks. The most often used counterparts used are ring and star networks, assuming complete degree homogeneity or heterogeneity, respectively. While these are important cornerstones, real networks have intermediate values with corresponding resistances that are still to be explored. Numerical simulations have been mostly based on Erdös-Renyi random graphs, whereas most real networks have much more heterogeneous and often scale-free degree distributions [19]. Furthermore, one relevant aspect of network topology has received very little attention in finance network theory: modularity. Modularity indicates how many links lie within a given community compared to the expected (random) links for a given link density of the network. High modularity (or similar measures such as clustering and compartmentalization) has been shown to act as a damper in epidemics spread [20,21] and propagation of extinctions in food webs [22]. In this respect, it is worth noting that the analytical foundations of the model we will describe below [23] show that, when the distribution of the shocks displays “fat” tails, extreme segmentation is optimal, while minimal segmentation and high density are optimal when the distribution exhibits “thin” tails. Globalization has drastically decreased the modularity of the financial network as the financial borders of national markets have vanished [24] and this can be sensed as a contributing factor to the dimension the 2007-2009 crisis had acquired.

Taking into account its implications on society, the available information on the effects of network topology on its shock-resistance is surprisingly scarce and fragmented. Furthermore, most of these studies have been carried out either categorically or varying one parameter at a time (OAT). However, OAT sensitivity analysis should only be used when the model is proven linear [25], as it misses interactions between model parameters and makes it difficult to assess their relative importance. The most famous example of interaction in network theory is the one that takes place between degree distribution and directedness of attack: Thus, it has been shown that in comparison with Erdös-Renyi networks, scale-free networks are more robust to random failure but more vulnerable to directed attacks [26]. 

Here we present a thorough study on contagion resulting from overlapping risk exposure, aimed to provide insights on the interplay of different network features. Our research starts from an analytical foundation and then explores the hyperspace of network topology features with numerical simulations. Finally, in order to make the connection with real world systems, we collect empirical data examples from the literature and assess the robustness of the core-group of the transnational corporate ownership network [27]. As will be shown below, our results provide important insights on hitherto unnoticed, joint effects of network parameters, insights that translate into relevant policy recommendations. 

## Model-Overview

**Figure 1:**
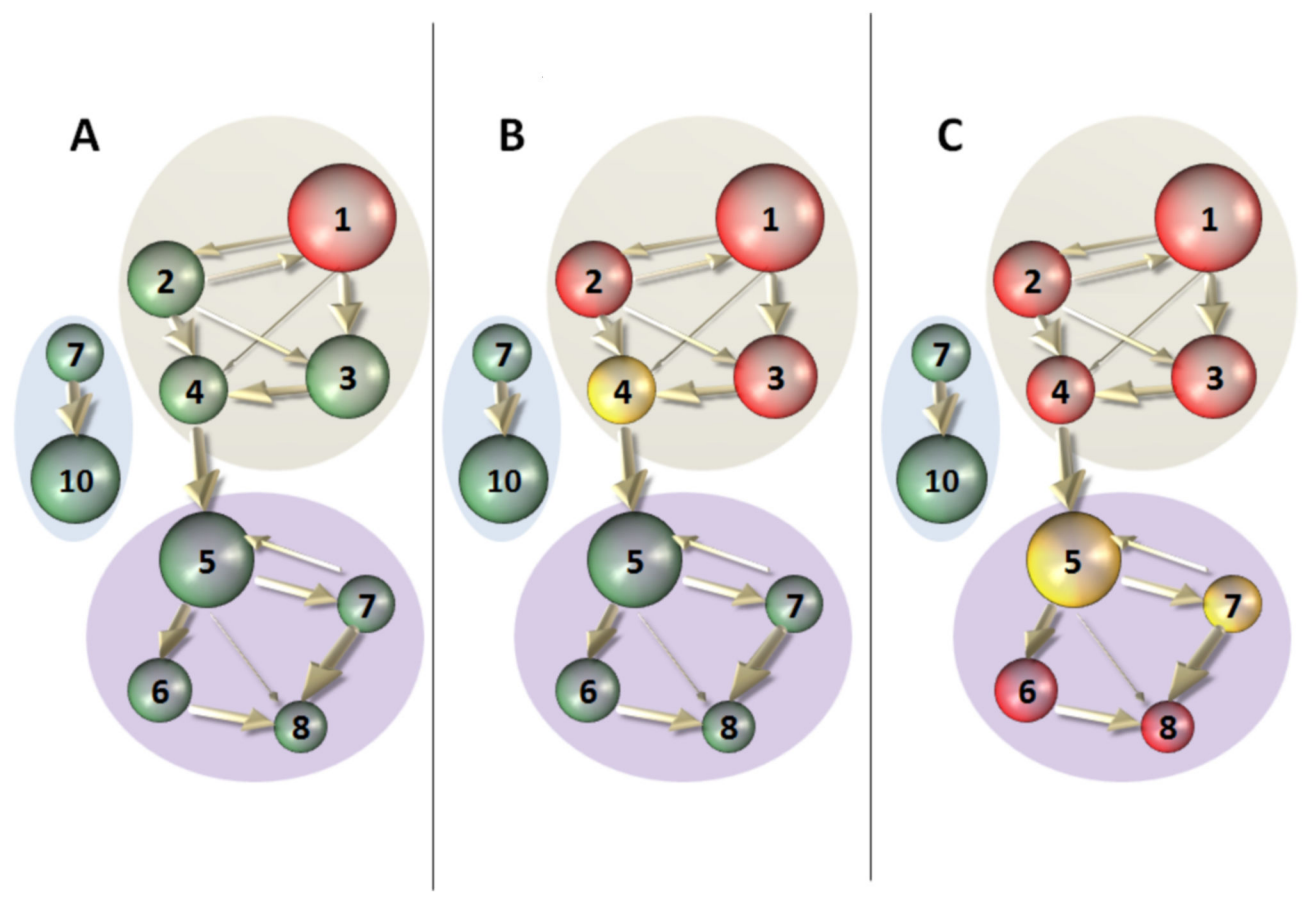
Schematic representation of the exposure network. Node size represents the asset volume of a firm, node color its level of capitalization (green: healthy, yellow: critical, red: default). Arrows thickness represents the amount of direct exposure. A) Firm 1 is hit by a relatively large shock and defaults. B) Firms 2 and 3 default due to their direct exposure to firm 1, the capitalization level of firm 4 drops to a critical level. C) Including the effect of indirect exposure, firm 4 defaults and the shock spreads until firm 8. Note that firm 5 and 7 can propagate the shock without having to default themselves. Nodes 9 and 10 are in an isolated cluster and are not affected.

We consider an environment with *N* risk-neutral financial firms (“banks”). At any given point in time, each firm has an investment opportunity – a project – which requires an initial payment (*I*) and yields a random gross return *R* at the end of the period. The resources needed to undertake the project are obtained by issuing liabilities (e.g. deposits or bonds) on which a deterministic rate of return must be paid. This means that, as the return on a firm’s investment is subject to random shocks, when the firm is hit by one such shock it may be unable to meet the required payments on its liabilities, in which case it must default (Figure 1). One important assumption of the model is that the largest possible shock will necessarily bankrupt all the participants of the network if they are connected at all. This will definitely hold with unbounded distributions.

A firm may benefit from entering risk sharing arrangements with other firms which allow it to diversify risks. The specific pattern of exchanges among firms is represented as a network, where a direct linkage between two firms reflects the fact that each firm holds a part of the asset of the other. Indirect exposure results from taking into account that a firm ends up having claims on the returns of projects of firms who hold assets of the firms it trades with, and so on. As a consequence a pair of firms lying at a certain distance in the network will have some reciprocal exposure to the yields of each other’s projects provided they are linked by a path through the network. 

The contagion process derives from the exposure of common assets losses; it is not a default-cascade with self-enhancing mechanism such as those described in {{607 Upper, Christian 2011}}. However, unlike most classical common exposure models, it does include indirect exposure, exemplified by this simply case: Firm A owns 50% of firm B who again owns 50% of firm C. If firm C is hit by a shock that reduces its value to cero, firm A would lose 25% of the original value of C. The process of asset exchange in our model is analogous to the transmission of pathogens in disease contagion. A “bad” asset (think of an eventually unpaid mortgage) will expose all common owners to a shock. This is transmitted from the originator bank to others who purchase its mortgage-backed securities, and further down the line to the bondholders of the companies which purchase those securities.

In an earlier theoretical paper [23], Cabrales et al. explored several cornerstones of the network structure of our model. A first variable is the size of the (disjoint) components into which the network is divided, i.e. the degree of segmentation of the system. A second dimension is the relative density of connections within each component. The third dimension is heterogeneity, opposing a star-like network to a regular network. The work we present in this paper allows us to extend significantly this model by exploring the parameter space between these extremes, to distinguish between size- and degree- heterogeneity and to assess the effect of capitalization level, going much further than what is amenable within analytical approaches. 

Throughout the whole paper, the term “optimal” is referring to the least number of mean defaults. 

## Results

### Analytical results

For the sake of completeness, we find it important to give a brief summary here of the main analytical findings about our model. The reader is referred to [23] for details. To begin with, when the probability distribution places high enough mass on relatively small shocks (“thin tails”), the best configuration has all firms arranged in a single and fully connected component. The main aim in this case is to achieve the highest level of risk sharing. Instead, in the opposite case where the probability distribution of the shocks exhibits “fat tails” (i.e., it attributes a high mass to large shocks), the optimal configuration involves a maximum degree of segmentation (that is, components should be of the minimum possible size). This reflects a situation where the priority is to minimize contagion. It is worth noting that these two polar cases, however, do not exhaust all possibilities. For more complex specifications of the shock structure (e.g. mixtures of fat and thin tails) intermediate arrangements are optimal, i.e. the optimal degree of segmentation involves medium-sized components*.*

Regarding heterogeneity, the main finding is that heterogeneity tends to favor assortative matching, that is, firms facing similar shock distributions should band together. This does not necessarily mean that they differ because one type of distribution is necessarily more “risky” or more correlated than another one. They could be different without being ordered in a first or even second order stochastic sense. But the fact that they are different means that the optimal structure to deal with shocks could be different between the two of them, and mixing firms of different types would lead to inferior risk-sharing properties. That means, in practice, that some activities should be isolated from others, for instance by separating the banks’ retail and investment activities. In this respect, it was also found that the symmetric structure is optimal when the shocks are not too large (because this maximizes risk-sharing possibilities) while the star structure is optimal for larger shocks.

### Numerical Results

**Figure 2:**
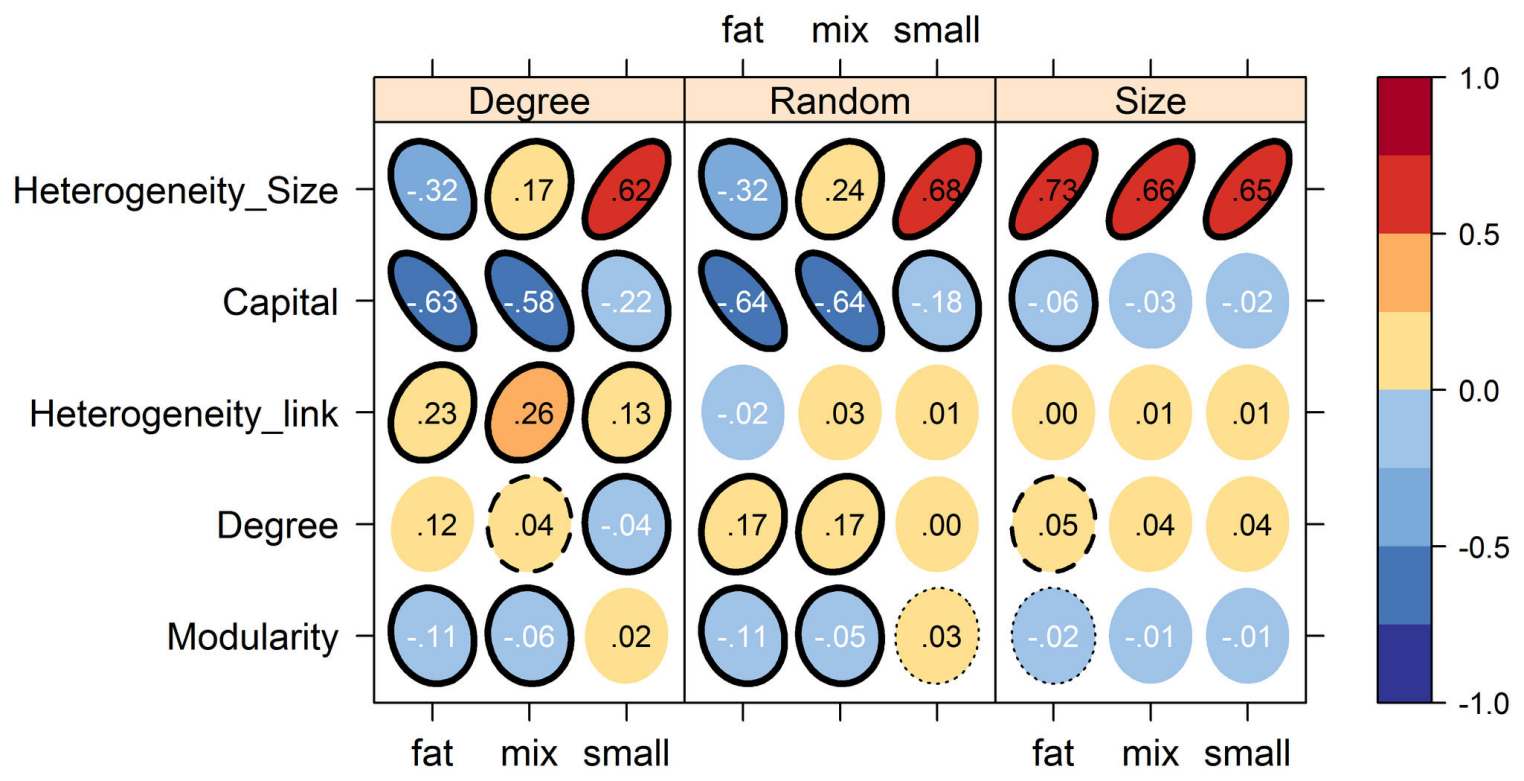
Standard regression coefficients (SRC) under different assumption on shock distributions. Shock sizes are sampled from a Pareto distribution with a scale parameter of either 0.5 (“fat”), 1.5 (“small”) or with 5% probability 0.5 and 1.5 with probability of 95% (“mix”). Shocks are either directed at the largest firm (“Size”), the most connected firm (“Degree”) or at a random firm (“Random”). Significance in linear regression is indicated by the border line, solid: p<0.01, dashed: p<0.05, dotted: p<0.1.

**Figure 3:**
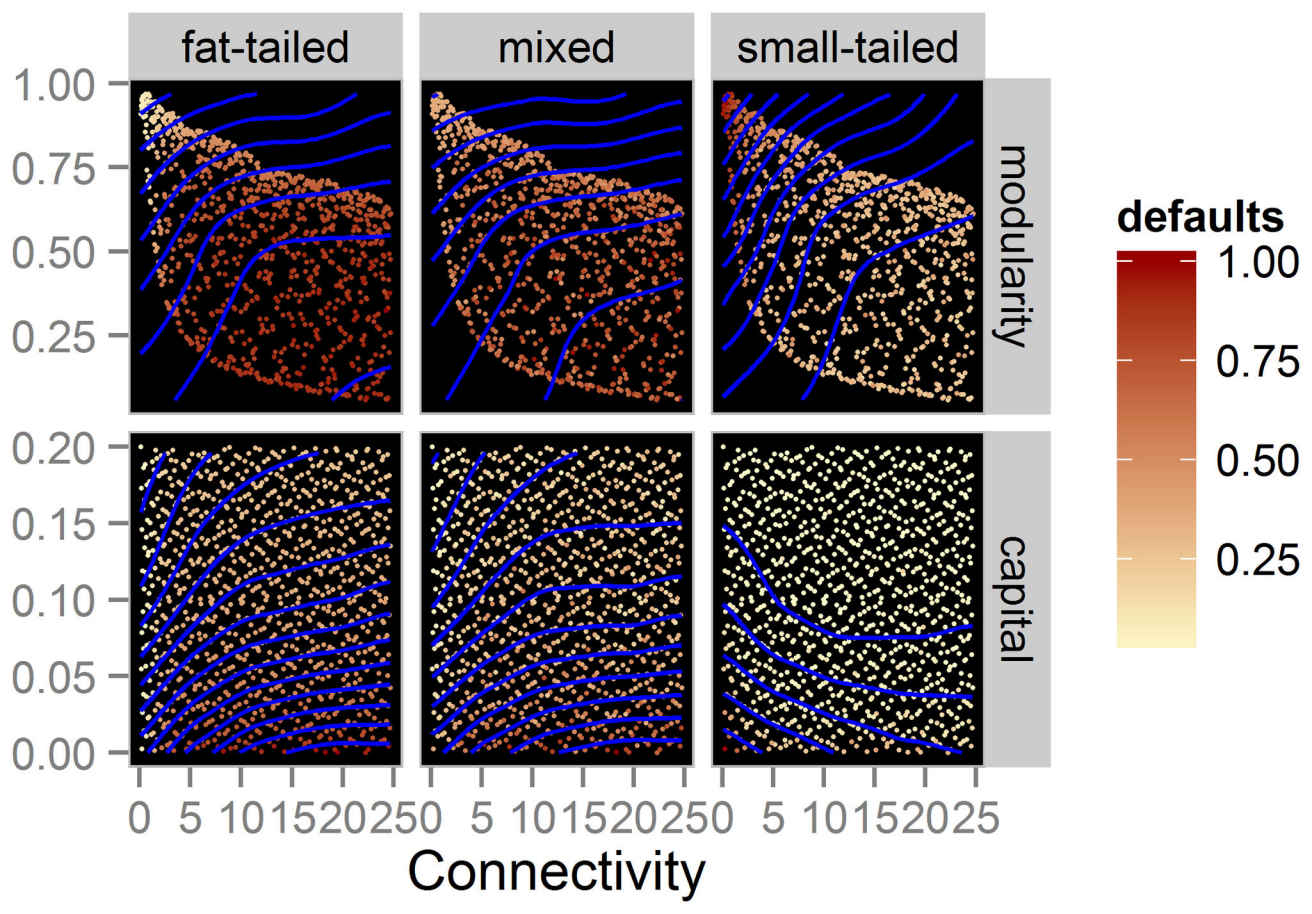
Effects of interactions on the number of mean defaults. The upper panel shows the interaction between connectivity and modularity, the lower panel the interaction between connectivity and level of capitalization. Blue contour lines represent the interpolation using a loess-function. Note that the number of mean defaults has been normalized for the range of each panel, and hence quantitative comparisons between panels are not possible.

Let us now present the main results of our paper, namely those obtained from our numerical simulation program (see Materials and Methods below for details on our procedure and analysis). Figure 2 summarizes the analysis made through a regression of our Monte Carlo simulations. As can be seen from the plot, a first conclusion is that the level of capitalization stands out as the most important individual parameter affecting network resistance to external shocks (Figure 2). It sets a clear upper limit to the mean number of defaults, which decays sharply for low capital requirements (maximum default is halved with ~10% capital requirement). However, the amount of capital required to achieve a given number of mean default depends greatly on network properties (Figure 3) and low mean default values are also obtained with very low capital requirements (see Figure S3).

The trade-off between diversification and contagion found in the analytical results could be confirmed and extended to other parameters. When shocks are sampled from a small tailed distribution, the optimal structure is well connected and uniform: default probability increases with the number of connections and decreases with modularity and size heterogeneity. But when shocks are sampled from a fat tailed distribution, the optimal structure is the opposite, sparse and heterogeneous (Figure 2). The effect of size heterogeneity, however, is highly depending on the shock target: when shocks are directed preferentially at the biggest firm, high heterogeneity enhances vulnerability independently of their size distribution. Surprisingly, degree heterogeneity increases vulnerability only when attacks are directed at the most connected firm and has no effect otherwise. Importantly, all of these results were robust to other statistical analysis techniques such as partial correlation coefficients and rank transformation.

**Figure 4:**
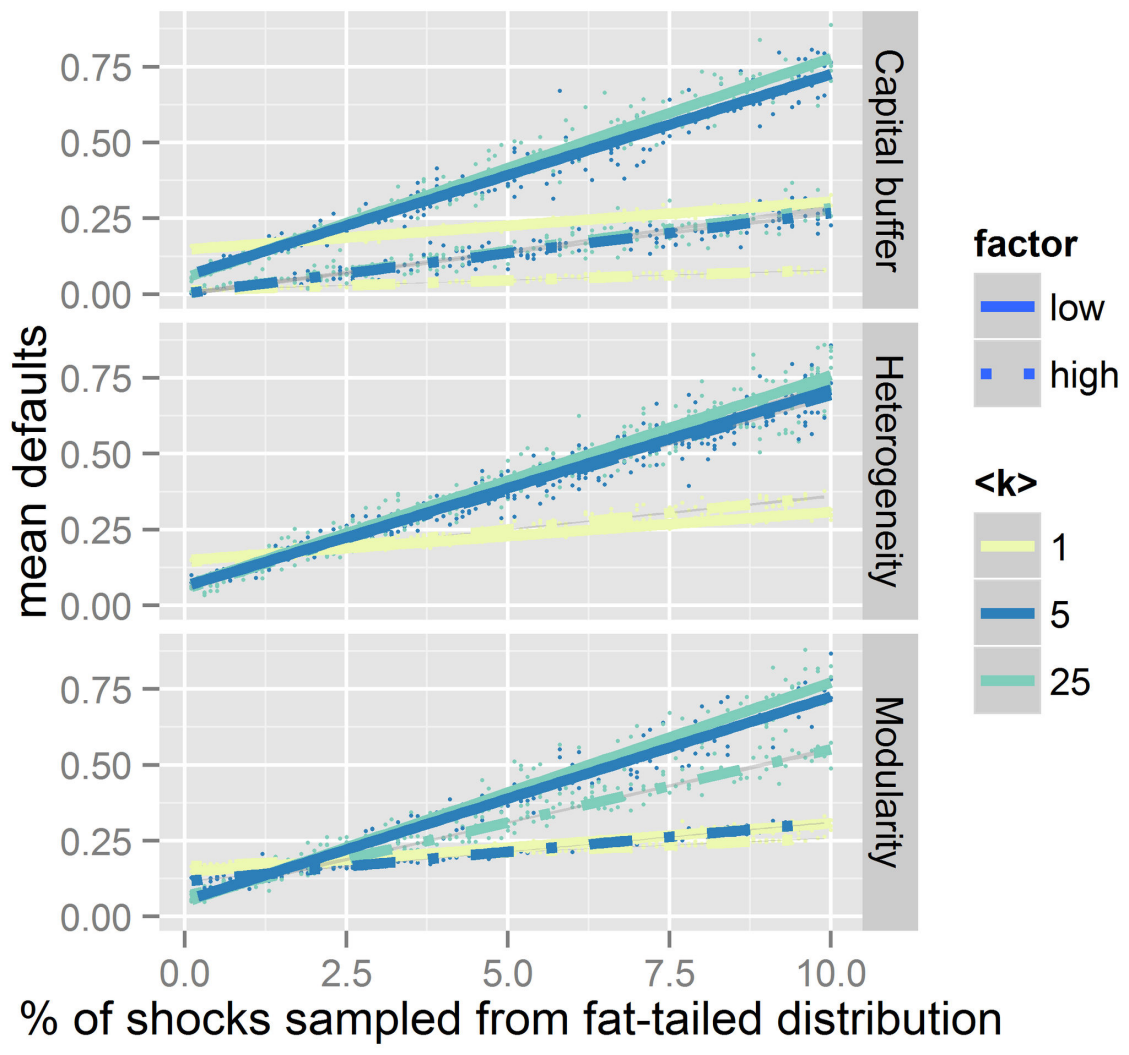
Mean defaults as a function of the fractions of shocks sampled from a fat-tailed distribution. The line colors represent different levels of mean degree (<k>).Continuous lines represent the trends for random graphs without capital buffer, Dashed lines represent the effect of the applied modifying factor: a 10% capitalization level (top), the highest possible degree heterogeneity (middle) and the highest possible modularity (bottom).

All response curves present high concavity. Once a given degree of density, modularity and heterogeneity is reached, further changes of these parameters result in little variation of default probability (Figure S3, S4 and S5). Accordingly, the variance explained by each parameter was improved when using rank transformations in most cases (see Figure S6). Important non-linearities resulted also from the interaction between parameters. For instance, modularity had no effect on mean default values when density was very high, as all nodes are effectively connected, or very low, as most nodes were isolated anyway (Figures 3 and 4, top panels). Similarly, the number of defaults increased much more steeply with density when modularity was low. Important non-linear interactions were also observed between size heterogeneity and density. The number of defaults could be limited by both density and level of capitalization (Figure 3). 

With constant network topology, default probability could be predicted from the fraction of shocks sampled from a fat-tailed distribution. The relationship was linear and the slope increased with density (Figure 4). An intersection point existed (approximately a 2% sample from fat-tailed distributions for random-graphs) where link density did not affect systemic risk. For fractions of fat tailed shocks below this value, risk diversification was beneficial, whereas above this level it turned out to be detrimental. Higher capital requirements lowered the intercept of the curves. A high modularity drastically reduces the slope at intermediate densities, but once more there was little effect for very dense or very sparse networks. Degree heterogeneity had no significant effect on slope or intercept (Figure 4).

**Figure 5:**
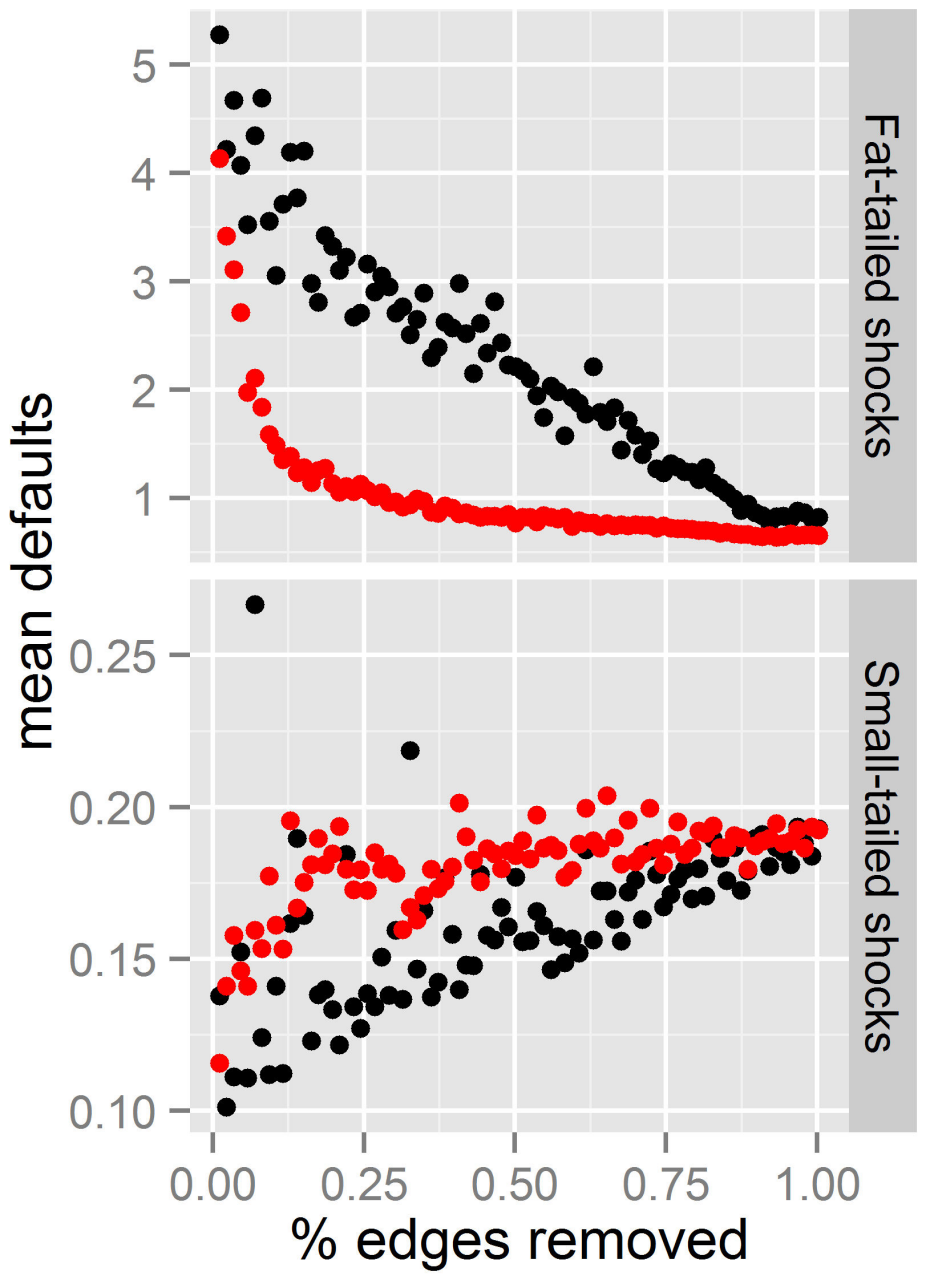
Default probability as a function of the number of edges removed edges from the core of the network of corporate ownership. Removal was done randomly (black squares), or preferentially at the edges with the highest betweeness centrality (red circles) or the highest degree heterogeneity (grey diamonds). Shock were sampled from a small-tailed Pareto distribution (σ=1.5, lower panel) or a fat-tailed Pareto distribution (σ=0.5, upper panel).

After completing the numerical study summarized above, we moved towards a closer connection with real systems by considering the core-group of the transnational corporate ownership network [27]. Thus, we studied the effect of removing links in the network, which implies severing ownership relations among firms. In this manner, we found that the relationship between vulnerability and the number of removed connections was linear when they were removed randomly or with preference for the links that contributed most to heterogeneity. The slope was negative when shocks were sampled from fat-tailed distributions and positive under small tailed shock distributions. However, when the links with the highest betweeness centrality were preferentially selected for removal, the decay was exponential (Figure 5) with fat-tailed shock distributions. 

## Discussion

The numerical results obtained here support and extend significantly the underlying analytical findings of [23]. Our simulation program allows us to better understand the trade-off between diversification and contagion, which we have seen holding for a large variety of networks. We also showed that the transition from safe to risky regimes can be very sharp en two aspects: 1) a small fraction of shocks sampled from fat-tailed distribution can easily change the optimal structure from well connected to isolated and 2) a critical range exists where adding or rewiring a few links has disproportionate effects on the mean number of defaults. This range is situated at low link densities and high values of modularity and size heterogeneity. Crucially, all real world examples found in the literature show precisely these characteristics. Since establishing connections is costly, real world networks tend to be rather sparse (see Table S2). Yet they are not immune to shock propagation: For instance, the network density measured in [28] was a very sparse 3 percent, but all nodes had a 97 percent influence domain, meaning that no matter where a contagion starts, it could reach nearly all banks. 

As interconnectedness allows for higher risk diversification, many studies e.g. [7,29,30] reach the general conclusion that banks are most exposed to systemic risk when density is low, and most resistant in a complete network. But interconnectedness is a two edged sword, as it can exaggerate the magnitude of default cascades. The combination of beneficial and adverse effects can lead to a non-monotonic relationship with an optimum at intermediate connectivity [9,17,31,32] as diversification gains are often more than offset by the costs of increased exposure to volatile activities [33] . The location of this optimum depends on the expected shock size distribution; both analytical work [23] and our results in Figure 4 show that the optimal density depends on the fraction of shocks that result from fat-tailed distribution. We therefore have to distinguish the vulnerability to financial distress in response to normal-sized shocks from that to large shocks [34]. The resulting incentives for the market left on its own are procyclical, as diversification increases the default probability of the banking system in case external assets pay a negative cash-flow (downturn) and decreases the default probability in case of positive cash-flows (upturn) [35]. This can be observed in the example of the global bank network [36]: the density of the network steadily increased during the “good times”, tending towards a small-tail shocks equilibrium that resulted to be dangerous in the “bad times” with fat-tailed shock distribution (see Table S2). Albeit density came down rapidly after the 2007-2009 crisis, the damage was already done. An analytical stress test [37] showed that the size of the default cascade generated by a macroeconomic shock across balance sheets may exhibit a sharp transition when the magnitude of the shock reaches a certain threshold. Here we show that even the qualitative response of the system changes when as few as 2% of the shocks are sampled from a fat-tailed distribution (Figure 4). However, it is almost impossible to distinguish one distribution from another in practice (see Figure S5). Note that since in our model contagion results only from shared asset losses, the existing firm network can be maladapted to the underlying economic uncertainty even without taking into account the different channels of shock amplifications [38], or conflicts between individual and systemic risk [39], but simply by a “black swan” [40] effect.

Considering the risk exposures that result from indirect neighbors greatly enhances the “effective” connectivity of the network. This accentuates the effects of the parameters that influence segmentation. An infinitely big shock could bring to bankruptcy every firm that can be reached by a path through the network. Real world shocks are not infinite but neither is network size and shocks can be several orders larger than the level of capitalization of many firms. This leads to a percolation phenomenon: when a giant cluster forms, almost all firms are susceptible to large shocks. Higher modularity increases the density at which the giant cluster forms and therefore decreases systemic risk.

Heterogeneity in degree distribution has received much attention in the literature during the last decade. In line with the seminal results of [26], our model network was more vulnerable to attacks directed at the most connected nodes. Degree heterogeneity is a crucial parameter in networks of infections as pathogens spread much faster and are more likely to become epidemics in scale-free than in random or regular networks. However, in our model, when attacks were not directed at highly connected nodes, no change in resistance could be observed. Note that our model does not simulate default cascades; stress can propagate through nodes even if they do not default. The most comparable statistics from epidemic would be epidemic size, an outcome much less sensitive to degree heterogeneity [41]. This has also important policy implications: higher capital requirements on systemically important financial institutes (SIFIs) might not prevent the spread of all shocks. Using again the analogy of epidemiology, one cannot tame the super-spreaders [6] by avoiding their infection, if they can propagate the disease without noticing the illness. This result also shows the importance of global parameter analysis: as a side effect, higher degree heterogeneity reduces the density at which a giant component forms; however, this effect vanishes when analyzed together with modularity which directly aims at the crux of the matter. 

Higher size heterogeneity can be considered as a different way of isolation since it leads to a more unequal distribution of the number of defaults. Consider the extreme case where nearly all of the investments are realized by one single firm connected (directly or indirectly) to many very small firms; In a regular network a large enough shock would result in the default of most firms, independent of which firm was hit. In a heterogeneous network no firm defaults when a small firm is hit which is much more likely given their larger number. Therefore size heterogeneity provides safety in the presence of large shocks. On the other hand, small shocks that would not result in any default under a regular size distribution can lead to a default of all firms when the biggest firm is hit. However, this effect is important only for very extreme heterogeneity and is only socially beneficial under the assumption of linearity between number of defaults and system cost [39]. If this function is assumed to be convex the optimal degree of size heterogeneity depends on the shape of this function. Our results are in line with [42] as a combination of high density, low modularity and similar sized firms leads to homogeneity in risk exposure. Connectivity and homogeneity have also been pointed out as early warning indicators of system collapse in finance and other networks [43]. Also complexity of financial derivates is now seen as a risk factor [44]. 

Our results confirm that capital requirements are an important tool (at least when the requirement are actually fulfilled and not eluded with accounting tricks), however the level of capital requested should depend on macro-prudential criteria. In that sense the Basel II and III accords establish higher capital requirements for SIFIs. The analysis of different national banking systems has shown that the SIFIs not necessarily are the biggest firms. Also, local measurements, such as number of links of a financial institution, are insufficient [45], as the magnitude of the shock propagation depends on the structure of the whole network, and identifying most central ones [5,11] is a much more promising approach. However, the knowledge of topology of the financial network is very fragmented and incomplete. When data is available, it is generally confined within one country. The global financial network is not homogeneous, as it has grown in segmented sub-networks limited by national borders and other historic reasons [46]. Globalization has increased the links between these national and functional clusters [47], reducing the modularity of the network. Again, this is a “natural” adaptation when shocks are supposed to come from a purely small-tailed distribution. However, this builds bridges that have allowed the financial crisis to become global. In connection with this, our study of the empirical data shows that the stability of the core enterprises of transnational corporate control could be greatly enhanced removing a small fraction of its links, if choosing the ones with the highest betweeness centrality values (Figure 5). If only local information on bank connections is available, we therefore recommend that the externality produced from links between countries or sectors should be taxed. As pointed out in [42], the Volcker rule in the United States, quarantining risky hedge fund, private equity and proprietary trading activity from other areas of banking business, is one example of enhancing modularity in practice. Our results imply that macro-prudential policies should target not only the firms, but also the links between them. This supports the idea of a “systemic risk charge”, where financial institutions pay into a fund proportional to their contribution to systemic risk. This is a complementary, not opposed, measure to increase capital requirements on SIFIs which might prove most relevant in preventing future crises.

## Materials and Methods

We consider an environment with *N* risk-neutral financial firms and a continuum of small investors. At any given point in time, each firm has an investment opportunity - a project - which requires an initial payment *I* and yields a random gross return *R* at the end of the period. It is assumed that the firm invests all the available funds besides a capital buffer. The resources needed to undertake the project are obtained by issuing liabilities (e.g. deposits or bonds) on which a deterministic rate of return must be paid.

The gross return of the project is random, as with some probability *q* the firm is hit by a negative shock. If no shock hits, the return equals some normal level *R*. The loss *L*_*b*_ is a random variable, with a Pareto distribution function.

Since the return on a firm's investment is subject to shocks, while the return promised to its creditors is deterministic, when the firm is hit by a shock it may be unable to meet the required payments on its liabilities, in which case it must default. Default costs are assumed to be substantial, so that the value of a firm is maximized when its probability of default at any point in time is minimized.

There is a large set of investors, who are the source of the supply of funds to firms. Investors are risk neutral and require an expected gross rate of return equal to *r* in order to lend their funds in any given period. Since firms may default, in which case creditors receive a payment equal to zero, the nominal gross rate of return *M* on the deposits to the firms must be greater or equal than *r*. Specifically, if we denote by φ the ex ante probability that any given firm defaults (an endogenous variable), we must have: 

$$M=\frac{r}{1-\phi }$$ (1)

Since, as stated above, default entails a significant cost for a firm, a firm may benefit from entering *risk sharing arrangements* with other firms and hence diversify risks. Here we consider the case where these arrangements take the form of shares of assets between firms, that is, of claims to the yields of the firms' investments, prior to the realization of the uncertainty. The possibly iterative procedure through which each firm exchanges shares on its whole array of asset holdings can be viewed as a *securitization* process of the firms' claims.

More precisely, let us posit that each firm exchanges a fraction 1-ϕ of its standing shares, giving rights to the return on its investments, for shares held by other firms. The specific pattern of exchanges among firms is formalized by a network, where a direct linkage between two firms reflects the fact that they undertake a *direct* exchange of their assets. These exchanges are symmetrical in the analytical model where all N firms are identical *ex ante*. However in the numerical model this symmetry is broken, supposing that the shares have been issued against a value that is not necessarily present in the system anymore, i.e. we just represent a present state without reconstruction the history that lead to it. We allow for these asset swaps to occur repeatedly. *Indirect* connections are then also formed. In the analytical model, these asset swaps are allowed to occur, whereby a firm ends up having claims on the returns of projects of firms who swapped assets with the firms it exchanges assets with, and so on. As a consequence a pair of firms lying at a certain distance in the network will have some reciprocal exposure to the yields of each other's projects provided the number of exchange rounds is high enough - in particular, as high as their network distance.

In the numerical model, we represented the financial network of exposures as a directed weighted graph that was constructed by a variant of the preferential attachment algorithm. We started from a given set of nodes (firms) connected with a few (~1%) random edges between them. Subsequent links were added until obtaining the desired network characteristics repeating the following algorithm for each link:

- Calculate current network properties (modularity and heterogeneity)
- Compare these results to the desired network properties
- Assign probabilities to each pair of (unconnected) nodes in order to reduce distance between actual and desired properties.
- Sample link from these probabilities

A more detailed description is given in Materials S1. Once the links between nodes are established, their firm sizes are sampled and assigned to the nodes taking into account the correlation between firm size and number of links. A fraction of the operational result (*f*_*E*_) is assigned to an external entity, emulating private investors whose default would not affect systemic risk. The rest is split evenly between the original node and all its neighbors. The result is an adjacency matrix A, where each entry *a*_*ij*_ represents the fraction firm *i* holds of the investment results of firm *j* (*r*_*j*_). 

To calculate the overall exposure we followed [48] and expressed the value *V*_*i*_ of firm *i* as:

$$V_{i}=\sum_{k}D_{ik}r_{k}+\sum_{k}a_{ij}V_{j}$$ (2)

where the matrix *D* equals zero in all entries but the diagonal ones, that take a value 1-external control (*f_E_*). Equation 1 can be written in matrix form and solved for *V* as:

$$V={\left(1-A\right)}^{-1}Dr$$ (3)

The values of *r* are obtained from the firms investments, that is their size (*S*_*i*_) minus their capital buffer (*K*_*i*_) and a risk adjusted return rate *q*. Some firms are hit by a shock (l) sampled from a Pareto distribution. 

$$r_{i}=(S_{i}-K_{i})•(q-l_{i})$$ (4)

The Pareto distribution has a fat-tail when its shape parameter (γ) is <1 and a small tail otherwise. The shock can affect either one single or several firms and be directed either at the most connected, biggest or randomly chosen firms. The shock is limited to the total size of the affected firm (*l*≤1). A firm defaults if its value is below its level of capitalization.

### Monte Carlo simulations and analysis

We ran the model for 1,000 parameter sets that were structured as Sobol-sequences [49] to equally cover the sampling space (Table S1). For each parameter set we constructed 10 networks and exposed each to 1000 shocks for each shock distribution, resulting in a total number of model runs of 10^7^. Standardized regression coefficients (SCR) were calculated using the sensitivity package of the R-project [50]. 

### Topology indices

Degree heterogeneity was calculated following [51] as a function of the degrees of the nodes of each link. Modularity expressed the number of links than fall within a given community (cluster) compared to the expected (random) value [52]. Size heterogeneity has been expressed using the Gini coefficient [53], which corresponds to the ratio of the area of the Lorenz curve to the area below the diagonal. Details are given in the Materials S1. 

## Supporting Information

Figure S1 **Clustering distance calculated as the decimal equivalent of a bitwise XOR operation.**
(TIF)

Figure S2 **Screenshot of the graphical user interface of the model.**
(TIF)

Figure S3 **Scatter-plots of the mean number of defaults against all parameters varied in the Monte Caro simulations with attacks directed at the largest firm.** The blue lines indicate the trend line obtained from a loess smoothing function.
(TIF)

Figure S4 **Scatter-plots of the mean number of defaults against all parameters varied in the Monte Caro simulations with attacks directed at the most connected firm.** The blue lines indicate the trend line obtained from a loess smoothing function.
(TIF)

Figure S5 **Scatter-plots of the mean number of defaults against all parameters varied in the Monte Caro simulations with attacks directed at a random firm.** The blue lines indicate the trend line obtained from a loess smoothing function.
(TIF)

Figure S6 **Rank transformed standard regression coefficients (SRRC) under different assumption on shock distributions.** Shock sizes are sampled from a Pareto distribution with a scale parameter of either 0.5 (“fat”), 1.5 (“small”) or with 5% probability 0.5 and 1.5 with probability of 95% (“mix”). Shocks are either directed at the largest firm (“Size”), the most connected firm (“Degree”) or at a random firm (“Random”). Significance in linear regression is indicated by the border line, solid: p<0.01, dashed: p<0.05, dotted: p<0.1.
(TIF)

Table S1 **Range of model input parameters compared to the properties of the core of the corporate ownership network.**
(TIF)

Table S2 **Network properties of financial networks found in the literature.**
(TIF)

Material S1 **Detailed description of the network construction procedure, model use and data source.**
(DOCX)
