# Supplementary figures and images for: Towards a Proper Assignment of Systemic Risk: The Combined Roles of Network Topology and Shock Characteristics
Source: PLoS One. 2013 Oct 17;8(10):e77526. doi: 10.1371/journal.pone.0077526 (PMC3798718; doi:10.1371/journal.pone.0077526)

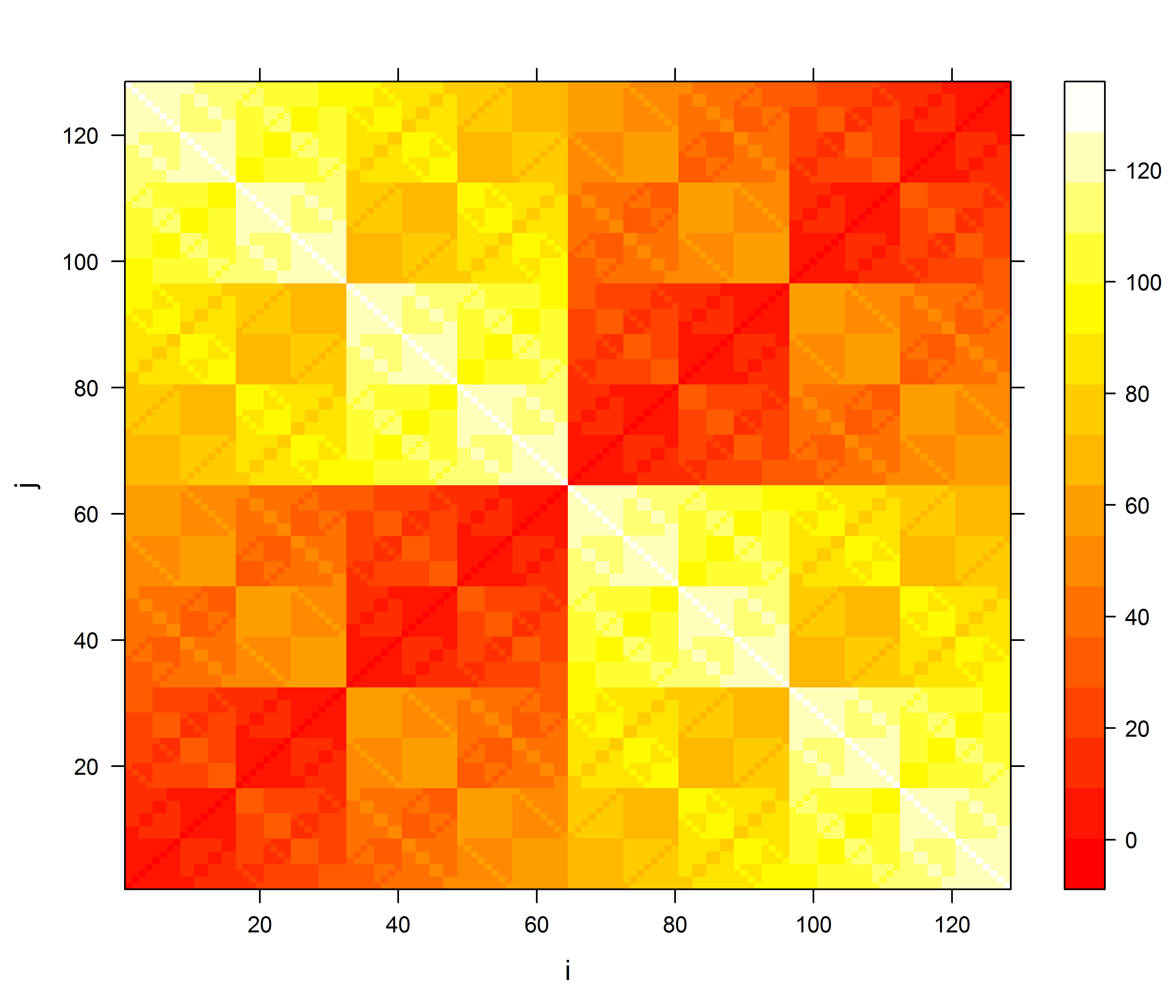

Supplement: Figure S1 — Clustering distance calculated as the decimal equivalent of a bitwise XOR operation. (TIF) [file pone.0077526.s001.tif]

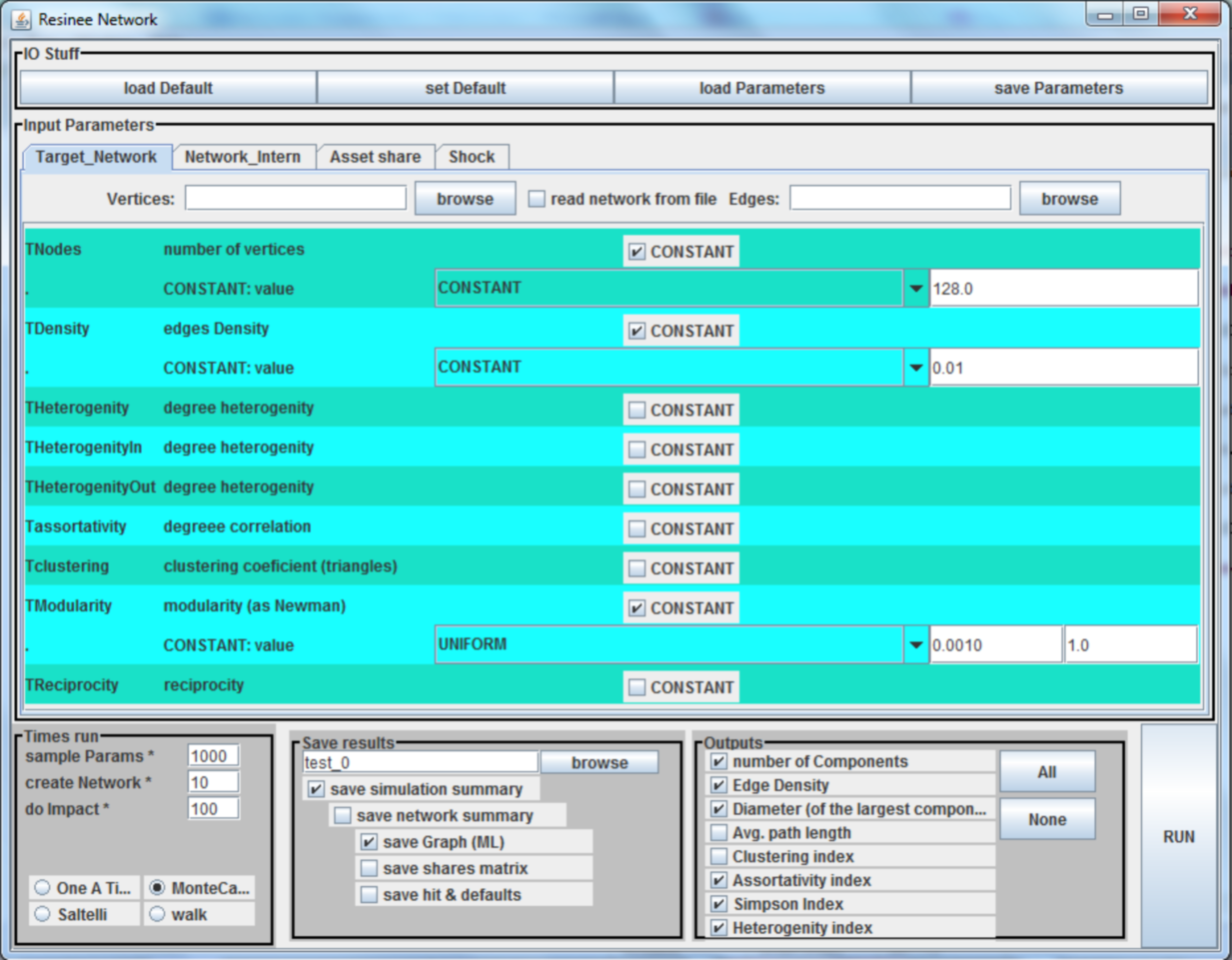

Supplement: Figure S2 — Screenshot of the graphical user interface of the model. (TIF) [file pone.0077526.s002.tif]

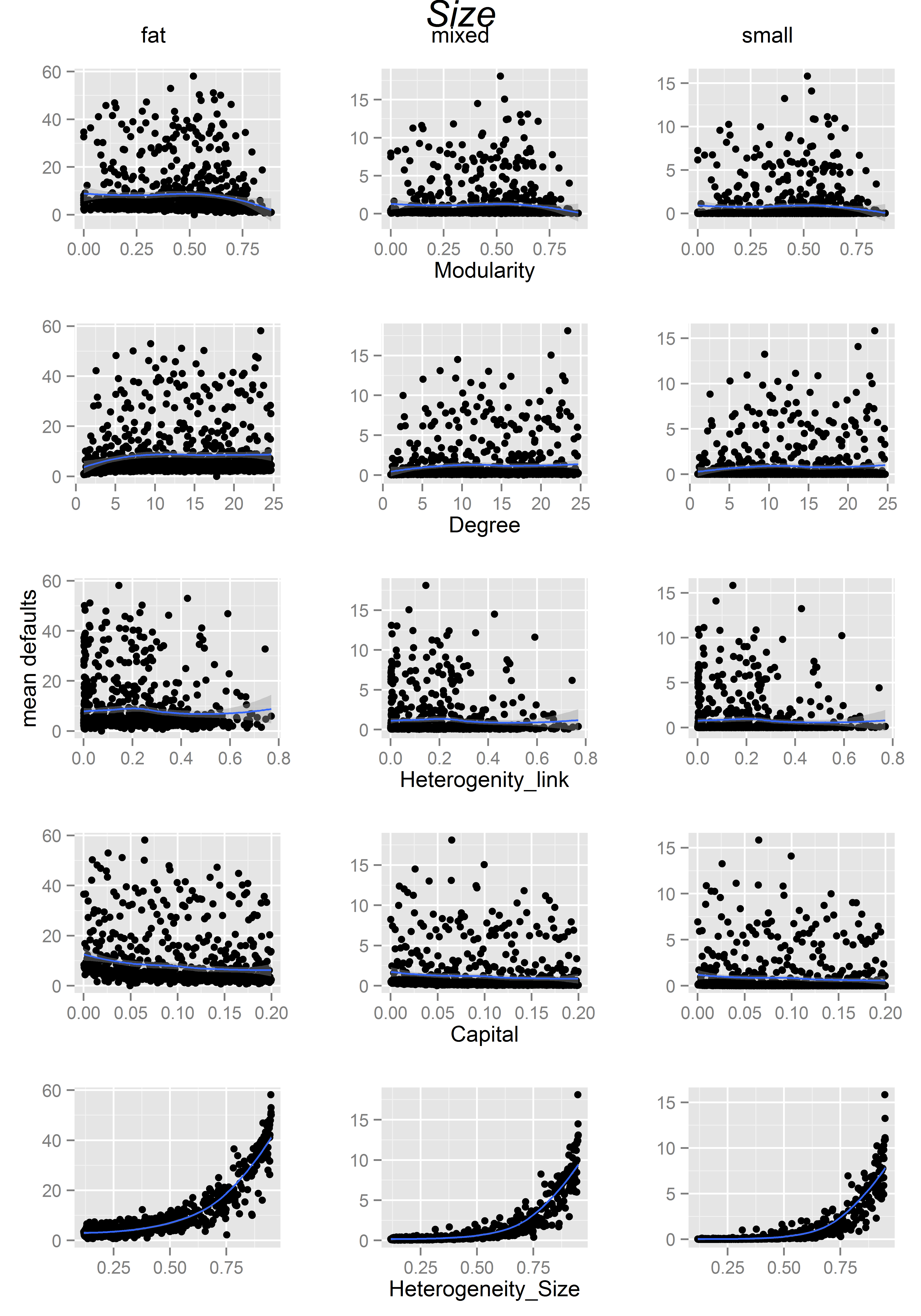

Supplement: Figure S3 — Scatter-plots of the mean number of defaults against all parameters varied in the Monte Caro simulations with attacks directed at the largest firm. The blue lines indicate the trend line obtained from a loess smoothing function. (TIF) [file pone.0077526.s003.tif]

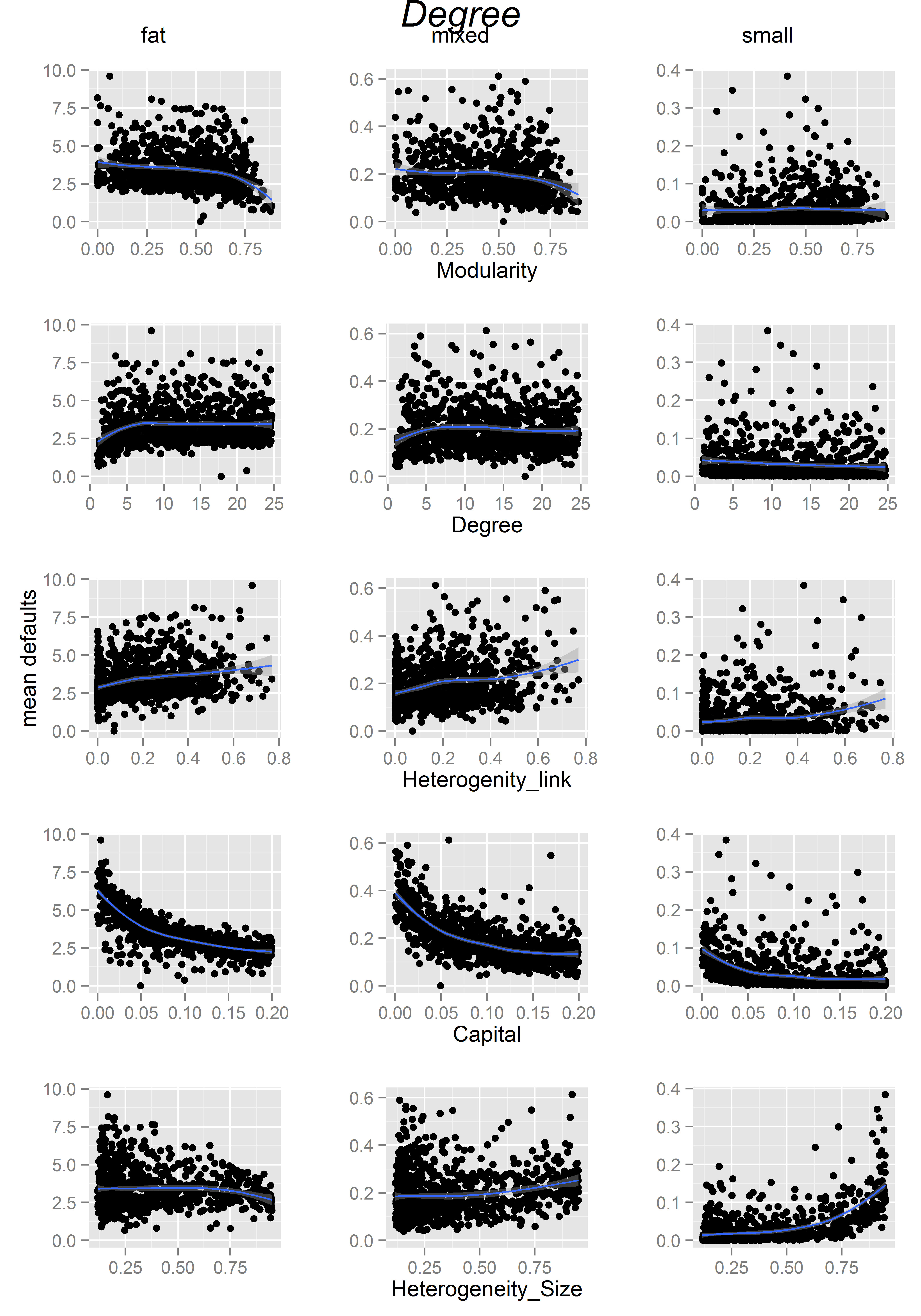

Supplement: Figure S4 — Scatter-plots of the mean number of defaults against all parameters varied in the Monte Caro simulations with attacks directed at the most connected firm. The blue lines indicate the trend line obtained from a loess smoothing function. (TIF) [file pone.0077526.s004.tif]

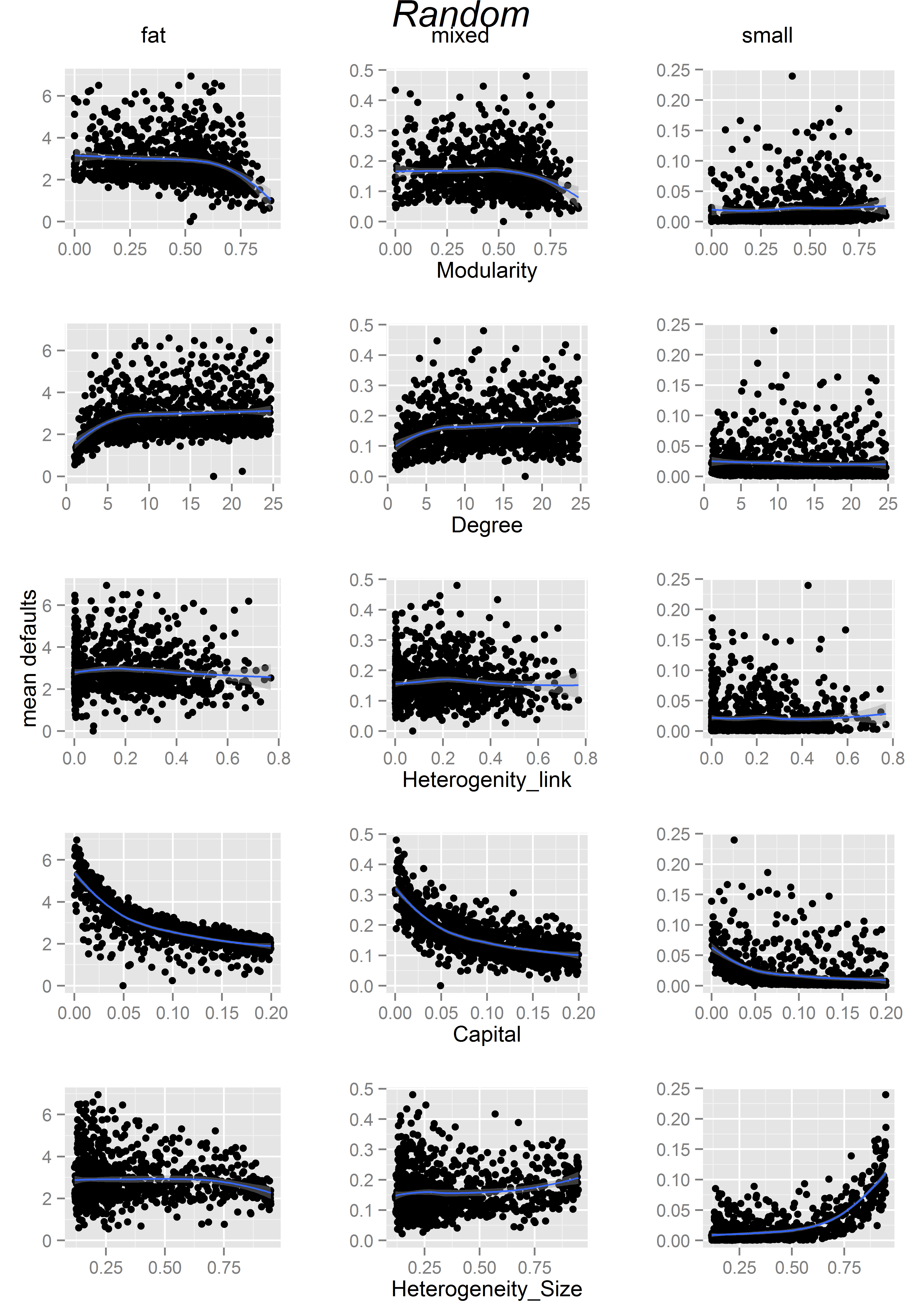

Supplement: Figure S5 — Scatter-plots of the mean number of defaults against all parameters varied in the Monte Caro simulations with attacks directed at a random firm. The blue lines indicate the trend line obtained from a loess smoothing function. (TIF) [file pone.0077526.s005.tif]

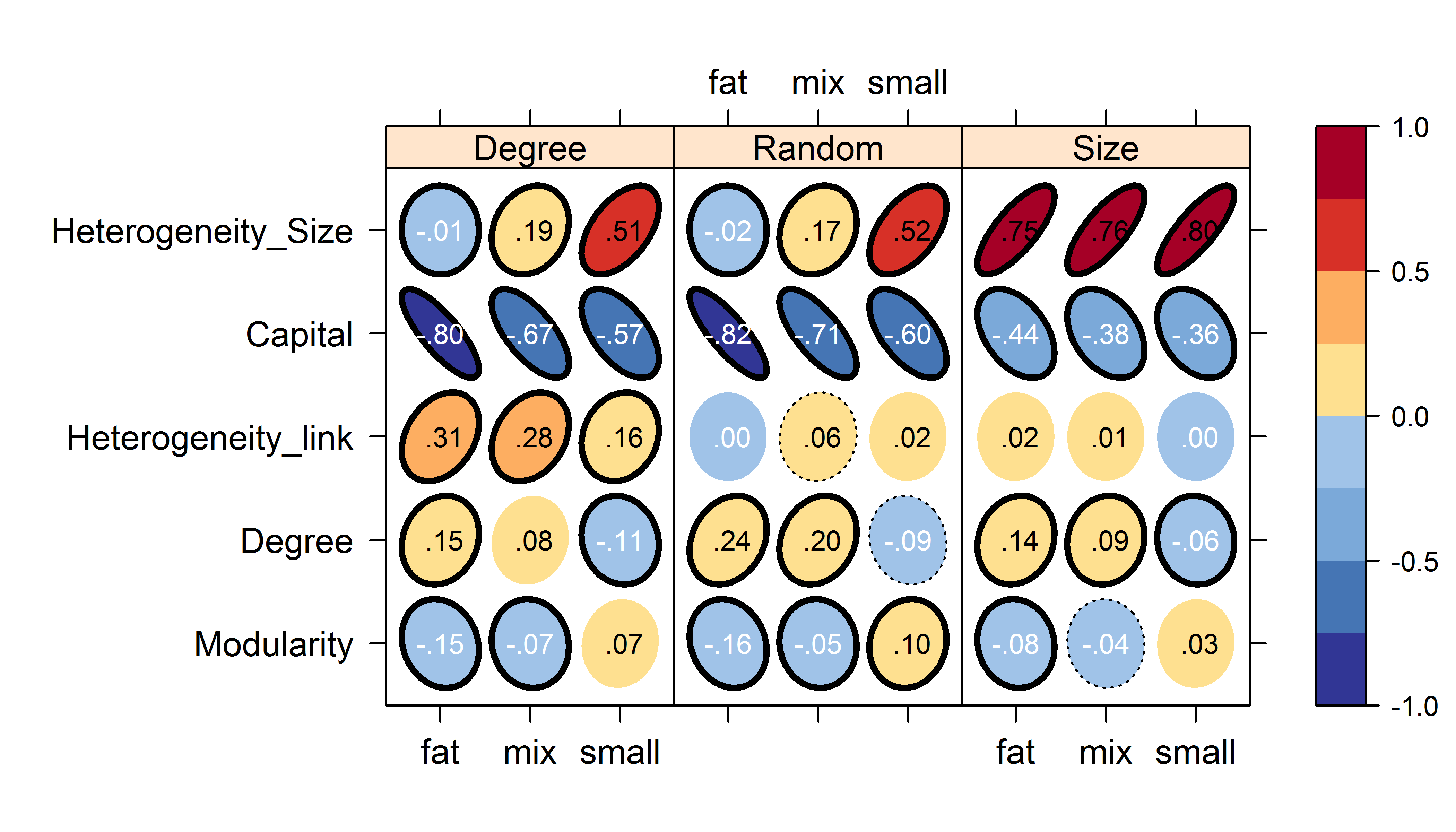

Supplement: Figure S6 — Rank transformed standard regression coefficients (SRRC) under different assumption on shock distributions. Shock sizes are sampled from a Pareto distribution with a scale parameter of either 0.5 (“fat”), 1.5 (“small”) or with 5% probability 0.5 and 1.5 with probability of 95% (“mix”). Shocks are either directed at the largest firm (“Size”), the most connected firm (“Degree”) or at a random firm (“Random”). Significance in linear regression is indicated by the border line, solid: p<0.01, dashed: p<0.05, dotted: p<0.1. (TIF) [file pone.0077526.s006.tif]
