# Supplementary material for: Towards a Proper Assignment of Systemic Risk: The Combined Roles of Network Topology and Shock Characteristics
Source: PLoS One. 2013 Oct 17;8(10):e77526. doi: 10.1371/journal.pone.0077526 (PMC3798718; doi:10.1371/journal.pone.0077526)
Supplement: Material S1 — Detailed description of the network construction procedure, model use and data source. (DOCX) [file pone.0077526.s009.docx]

# Supplementary Material for

**"Towards a proper assignment of systemic risk: the combined roles of network topology and shock characteristics."**

**by Lasse Loepfe, Antonio Cabrales and Angel Sánchez**

## Network construction

The financial network was represented as a directed weighted graph where the nodes represent the *financial* institutions (“firms”) and the links between them the ownership. We started to build the graph with all the nodes present and a few (1% of the desired number of edges) random edges. Each node was assigned an identification number, ranging from 0 to *N* (the total number of firms). Then the remaining edges to reach the desired network density were added following an iterative preferential attachment procedure:

1. Calculate the current topological indices of the network.
2. Calculate the effect that a new link would have on all topology indices for all potential links.
3. Assign link probabilities to all potential links according to steps 1 and 2 and sample the link.
4. Repeat 1-3 until desired network density is reached.

The topological indices were calculated as follows:

Degree heterogeneity was calculated following (1)as a function of the degrees of the nodes (*k*) of each link:

$$H_{R}=\sum_{i,j\epsilon E} (k_{i}^{-1/2}-k_{j}^{-1/2})$$

Modularity expressed the number of links than fall within a given community (cluster) compared to the expected (random) value (2):

The crux in modularity calculation is the detection of the community structure that maximizes modularity, which is a NP-hard problem. For the statistical analysis modularity has been calculated with the igraph library (3) of the R-project, using the walktrap-algorithm (4)for community detection. However, this approach was too expensive in computational time to use it within the network construction; therefore, it was calculated from pre-assigned clusters with density-dependent size.

The contribution of each potential link to heterogeneity was zero if the link already existed or *i=j*, and otherwise:

$$\text{Hp}_{\text{ij}}=\frac{(k_{i}-k_{j})^{ah*(H_{T}-H_{R})}}{\sum_{i} \sum_{j} (k_{i}-k_{j})^{ah*(H_{T}-H_{R})}}$$

Where *ki* and *kj* are the corresponding degrees of nodes *i* and *j*, *H_T_* the desired heterogeneity, *H_D_* the current heterogeneity and *ah* an even number scaling parameter.

The contribution of each potential link for modularity was zero if the link already existed or *i=j*, and otherwise:

Where “^” is the bitwise XOR Operator which copies the bit if it is set in one operand but not both and ID is a consecutive identification number ranging from 0 to NThis is a short way of establishing a hierarchical binary clustering tree. For instance, the binary equivalent of the decimal number “1” is “0000 0001”, of “2” “0000 0010”. Applying the bitwise XOR operator to these two numbers gives “0000 0011” or “3” in decimal numbers. So one can define the “clustering difference” as the decimal outcome of the bitwise XOR operator. The “clustering differences” for 128 nodes are plotted in supplementary figure 1.

The probability to be sampled is simply the sum of *Mp* and *Hp*. The sum can be weighted if one wants to give higher priority to either of the topology indices.

## The Use of the Model

The graphical interface allows the user to easily set the parameters of the model (supplementary figure 2). Some parameters apply to the network as a whole, e.g. network density. In others one can choose the distribution among firms, for instance firm size can be selected to be sampled from a normal distribution with mean x and standard deviation σ. The user can specify the number of simulations, as well as network- and shock- replications. If preferred, parameters can also be read from an external file. Also networks can be read from several standard file formats. The outputs include a summary file with a line for each parameter set, a “pajek” or “graphml” representation of the network, the adjacency matrix as a text file and a list of all shocks and their impact. The model is freely available on request.

## Corporate ownership data experiment

The data for the analysis of the network of corporate ownership was obtained from the Orbis data base and corresponds to the core of the bow-tie structure identified by (5). It containes 1318 nodes and 8577 directed links between them. From the original database it was not clear how many of the links represented direct or indirect ownership. In this experiment all links were treated as directed. Firm size (*I*) was set proportional to the reported operational revenue, or, when this was absent or negative, sampled from the distribution that best fitted the present data. When the weight of the outgoing links of one firm summed more than one, they were normalized. Links were removed in steps of 100, between which the network was exposed to shocks, the defaults counted and all values reset. When removing the links with the highest betweeness or heterogeneity, these values were calculated before each step.

References

1. Estrada E (2010) Quantifying network heterogeneity*. Physical Review E* 82(6): 066102.

2. Newman ME (2006) Modularity and community structure in networks*. Proceedings of the National Academy of Sciences* 103(23): 8577-8582.

3. Csardi G & Nepusz T (2006) The igraph software package for complex network research*. InterJournal, Complex Systems* 1695: 38.

4. Pons P & Latapy M (2005) Computing communities in large networks using random walks*. Computer and Information Sciences-ISCIS 2005* : 284-293.

5. Vitali S, Glattfelder JB & Battiston S (2011) The network of global corporate control*. PloS One* 6(10): e25995.

6. Soramäki K, Bech ML, Arnold J, Glass RJ & Beyeler WE (2007) The topology of interbank payment flows*. Physica A: Statistical Mechanics and its Applications* 379(1): 317-333.

7. Inaoka H, Ninomiya T, Taniguchi K, Shimizu T & Takayasu H (2004) Fractal network derived from banking transaction-an analysis of network structures formed by financial institutions*. Bank of Japan Working Papers* 4

8. Bech ML & Atalay E (2010) The topology of the federal funds market*. Physica A: Statistical Mechanics and its Applications* 389(22): 5223-5246.

9. Iori G, De Masi G, Precup OV, Gabbi G & Caldarelli G (2008) A network analysis of the italian overnight money market*. J Econ Dyn Control* 32(1): 259-278.

10. Vila Wetherilt A, Soramaki K & Zimmerman P (2010) The sterling unsecured loan market during 2006-08: Insights from network theory

11. Cajueiro DO & Tabak BM (2008) The role of banks in the brazilian interbank market: Does bank type matter?*. Physica A: Statistical Mechanics and its Applications* 387(27): 6825-6836.

12. Rørdam KB & Bech ML (2009) The topology of danish interbank money flows*. Finance Research Unit* 1

13. Hale G (2012) Bank relationships, business cycles, and financial crises*. J Int Econ*
